# The conceptualization of acute bronchitis in general practice – a fuzzy problem with consequences? A qualitative study in primary care

**DOI:** 10.1186/s12875-023-02039-z

**Published:** 2023-04-06

**Authors:** Nadine Schubert, Thomas Kühlein, Larissa Burggraf

**Affiliations:** 1 Friedrich-Alexander-university Erlangen-Nürnberg, institute of general practice, Universitätsstraße 29, 91054 Erlangen, Germany; 2 University of education, Department of sociology, Schwäbisch Gmünd, Germany

**Keywords:** Acute bronchitis, Respiratory tract infection, Antibiotic prescribing, Diagnostic uncertainty, Disease conceptualization, Primary care

## Abstract

**Background:**

Acute bronchitis is one of the most frequent diagnoses in primary care. Scientifically, it is conceptualized as a viral infection. Still, general practitioners (GPs) often prescribe antibiotics for acute bronchitis. The explanation for this discrepancy may lie in a different conceptualization of acute bronchitis. Therefore, we wanted to know, how GPs conceptualize acute bronchitis, and how they differentiate it from common cold and pneumonia. Furthermore, we tried to find out the GPs’ reasons for prescribing antibiotics in those cases.

**Methods:**

To answer our study questions, we conducted a qualitative study with GPs in Bavaria, Germany, by using semi-structured guided interviews. The analysis of the data was conducted using the documentary method according to Ralf Bohnsack. The transcripts were subdivided into categories. Analyzing each part by reflective interpretation, first manually, secondly with the help of RQDA, we extracted the most representative citations and main messages from the interviews.

**Results:**

The term acute bronchitis seems to be applied when there is neither certainty of the diagnosis common cold, nor of pneumonia. It seems it bridges the gap of uncertainty between supposedly harmless clinical pictures (common cold/viral), to the more serious ones (pneumonia/bacterial). The conceptual transitions between common cold and acute bronchitis on the one side, and acute bronchitis and pneumonia on the other are fluid. The diagnosis acute bronchitis cannot solve the problem of uncertainty but seems to be a label to overcome it by offering a way to include different factors such as severity of symptoms, presumed signs of bacterial secondary infection, comorbidities, and presumed expectations of patients. It seems to solve the pathophysiologic riddle of bacterial or viral and of decision making in prescribing antibiotics.

**Conclusion:**

Acute bronchitis as an "intermediate category" proved difficult to define for the GPs. Applying this diagnosis leaves GPs in abeyance of prescribing an antibiotic or not. As a consequence of this uncertainty in pathophysiologic reasoning (viral or bacterial) other clinical and social factors tip the balance towards antibiotic prescribing. Teaching physicians to better think in probabilities of outcomes instead of pathophysiologic reasoning and to deal with uncertainty might help reducing antibiotic overprescribing.

**Supplementary Information:**

The online version contains supplementary material available at 10.1186/s12875-023-02039-z.

## Background

Worldwide, upper, and lower respiratory tract infections are among the most recurring problems managed in primary health care. In Germany, acute bronchitis is the most frequent diagnosis applied in primary care practices when consulting a patient with cough [1]. Scientifically, acute bronchitis is conceptualized as a viral infection, affecting the bronchi, and producing its main symptom: Cough. As acute bronchitis is of viral origin mainly, in principle antibiotics are not recommended [2, 3]. The ailment seems to be more a variety of the common cold than a disease in its own right, with boundaries not being clearly defined [2, 4]. Classificatory, acute bronchitis is framed by the common cold on the one side and pneumonia on the other. The common cold is of viral origin and antibiotics clearly are not indicated [5]. In contrast, in pneumonia antibiotic prescribing is almost mandatory [6]. There is agreement that in respiratory tract infections antibiotics are prescribed too often in response to patients’ complaints [7]. However, not only the complaints of the patients play a role, but also reasons like the patient's expectations and the GP's assumptions of these expectations about it [8, 9]. According to literature, also their perception of the patient is a strong predictor for higher medical needs [10, 11]. Patients do not necessarily understand the harm of antibiotic overprescribing and can have misconceptions about diseases for which antibiotics are indicated [12]. Patients frequently put pressure on their GP towards antibiotic prescribing [13]. In addition, research has shown irregular findings in physical examination, different specialist pre-education and regional, geographic influencing factors relating to higher antibiotic prescribing [14]. GPs sometimes even indicated time constraints in every day practice as a reason to prescribe antibiotics [15].

Antibiotics do have small positive effects concerning the speed of clearing of symptoms [16]. Yet, as there are no significant differences to placebo concerning threatening outcomes, the indication for antibiotics needs to be considered in the broader context of potential side effects, medicalisation of a self-limiting condition, antibiotic resistance and costs [16]. According to national and international guidelines, the side effects outweigh the benefits, so antibiotics are not recommended for acute bronchitis. [3, 4] Antibiotic prescribing might be rational in certain circumstances, but the proportion of people receiving antibiotics for respiratory tract infections keeps being too high [17]. Compared to the European average, Germany is not among the top countries of antibiotic prescribing, but the prescription rate is still too high, especially for acute respiratory infections [18, 19]. A large part of antibiotics is prescribed by GPs [20]. If acute bronchitis is conceptualized as having a viral origin and any GP knows that antibiotics do not help against viruses, a different concept of acute bronchitis might be at work explaining why GPs keep prescribing antibiotics for it. Therefore, we wanted to know, how GPs conceptualize acute bronchitis and its border-categories, the common cold and pneumonia, how they discriminate between these three and where and why they see an indication for an antibiotic.

## Methods

To answer our research question, a qualitative study design based on semi-structured interviews was chosen. The interview strategy followed the concept of the so called “expert interview” [21]. In our case, the interviewer acted as a co-expert, which allowed us to have a symmetrical interaction at the same level concerning the research topic. This way, the interviewer could guide the GPs through the interview to extract knowledge about the research topic [21].

### Sampling

We followed a convenience sampling strategy, which turned out to be well balanced in terms of a variety of different GPs regarding age, sex, and practice location. We identified GPs in the surrounding of the cities Erlangen and Forchheim (Bavaria, Germany), using web search and personal contacts. These GPs were then invited via letter or phone. Teaching physicians on the list of the Institute of General Practice at the University Hospital Erlangen were excluded because we assumed that they might not be representative for the majority of GPs due to their academic affiliation and specific training.

### Interview guide

An interview guide (Additional file 1) was developed by the interdisciplinary team of researchers based on a literature review. The following topics were developed:

- The concepts of the common cold, acute bronchitis, and pneumonia with a focus on the differences between the three diagnoses
- The reasons of antibiotic prescribing or not-prescribing after diagnosing one of them

After the first three interviews, the interview guide was discussed and changed. The first interview guide contained too many closed questions; thus, we noticed a lot of breaks preventing fluent conversation with the interview partner. Therefore, questions were modified to be more open, and the personal perspective of the GP was added to extract the individual concept that might be existing behind. In addition, this created more space for the participant’s thoughts and reactions. Field-notes on remarkable reactions or important points of the interviews were taken immediately after the interviews and used to put what was said into an individual context, especially regarding nonverbal expressions or certain behaviors.

### Interviews

GPs were informed about the research topic and received an information sheet about the study at least two weeks before the interview took place. Questions of the GPs regarding the study itself were clarified before starting the interview. All GPs gave their informed consent to participate by signing a form. Before the interview was started, every GP filled in a demographic questionnaire (Additional file 2). The questionnaires were anonymized and used only to describe the sample. The interviews took place in the GPs’ offices and were audio-recorded digitally. Interviews were done in German. They lasted between 30 to 45 min each and were done between January and October 2018.

The interviews were transcribed according to Bohnsack’s transcription guidelines ‘Talk in Qualitative research’ [22, 23]. Due to process of transcription, data were treated on a numbered basis and thereby anonymized. Quotations in the manuscript were later translated into English thereby using help of a translation website [24].

### Data analysis

In order to reach the necessary depth of understanding the diagnostic and therapeutic reasoning, data analysis followed the documentary method of Ralf Bohnsack. This method allows to understand how the thought processes of the interviewees work, making it possible to distinguish between comprehension and interpretation of words [22, 23]. Therefore, it was considered best suitable for our study. Accordingly, the focus in our study was put on identifying contexts of meaning and the concepts associated with them. The transcripts were subdivided into main categories and subcategories, taking the key topics of the interview guidelines into consideration. This step was followed by the so called “phrasing interpretation” technique which is supposed to picture the apparent meaning of the spoken words [22]. According to Bohnsack, whenever a statement is given, there is more to hear than the words express. A spoken sentence is influenced by many things—the sound of voice, non-verbal action, or the speakers background and even the background of the conversation partner. Therefore, after transcription, we focused on this immanent meaning of the spoken word, the so called reflective interpretation [22]. By breaking the interviews down discursively and analyzing the subsections step-by-step, the methodology enabled us to reach a detailed interpretation of the text. Here, the results were discussed between NS [physician in vocational training] and LB [sociologist] and interpreted together. This enabled us to develop hypotheses from our research approach, the initially subjective conceptualization of the GP, that allowed us to look at the issue of antibiotic overprescribing from a new angle. To be consistent with our methodology, we wanted to stay as close as possible to data. Therefore, at first, no software or program was used for the interpretation. Instead, the interpretation of the interviews was done manually and documented in a tabular listing. In a second step, the software RQDA (version R i386 4.0.0) was used to make the keywords clearer, to re-examine their linkage to the passage, and to facilitate the selection of quotes from the interviews [25]. The results and the points of saturation were critically discussed and refined by the research team throughout the entire process. The GPs were not personally included in this process. If interest in our research findings was shown, it was recorded so that the GPs will get the article once it will be published. To revise the manuscript, the COREQ Checklist was used [26].

## Results

### Description of the study sample

The study population consisted of a sample of twelve GPs in Bavaria, Germany, most of them working in group practices. The GPs were aged between 30 and 70 years. More women than men took part in the study (Table 1).

**Table 1:** Results of the demographic questionnaire

Question content	*n* = 12	Percentage (%)
Office location		
------City radius 20 km	6	50%
------Rural area	6	50%
Form of practice		
------Single office	4	33%
------Group practice	5	42%
------Joint practice	1	8%
------Medical care center	2	17%
Number of colleagues when working in a group/joint practice/care center		
------1	1	8%
------2	2	17%
------3	0	0%
------4 or more	5	42%
Average no. of patient		
------ < 500	0	0%
------500–1000	2	17%
------1001–1500	3	25%
------ > 1500	6	50%
Sex of the GP		
------Male	4	33%
------Female	8	67%
Year of birth (GPs)		
------ < 1950	1	8%
------1950–1960	1	8%
------1960–1970	6	50%
------1970–1980	2	17%
------ > 1980	2	17%
Year of establishing the GP’s office		
------Before 1980	2	17%
------1980–2000	2	17%
------2000–2010	3	25%
------ > 2010	5	42%
Specialty		
------GP	9	75%
------GP Internist	3	25%
------Others* (Emergency, Sport, Chiropractic)	4	33%

^*^Additional specialties, GPs who have an additional designation besides GP or GP internist

### Categories applied to the transcripts

There were three main categories with their subcategories derived from the questions of the interview guideline (Table 2):

**Table 2:** Main categories and subcategories regarding conceptualization of the acute bronchitis

*Main categories*	Subcategories
***1.Acute bronchitis, common cold and pneumonia: Concepts and differentiation***	*Conceptualization of acute bronchitis*
	*The common cold and its differentiation from acute bronchitis*
	*Pneumonia and its differentiation from acute bronchitis*
	*Role of technical diagnostics*
	*Diagnostic uncertainty and gut feelings*
***2. Patients’ expectations concerning therapy***	
***3. Antibiotic prescribing in respiratory tract infections***	*The concept of secondary bacterial infection*

1. ***Acute bronchitis, common cold and pneumonia: Concepts and differentiation***

Starting with the concept of acute bronchitis, the concepts of the two adjacent categories common cold and pneumonia were derived. The subcategory "Role of technical diagnostics" arose especially in the conversation about pneumonia and related mainly to laboratory tests (blood samples) and X-rays. The last subcategory “Diagnostic uncertainty and gut feelings” was present in all sections of the interviews but particularly linked to the decision of antibiotic prescribing. In addition to that, even before being asked for, antibiotic prescribing was always mentioned as a particularly important point by the GPs themselves.

#### Conceptualization of acute bronchitis

Acute bronchitis was seen as a category “in between” without clear signs or borders, where the patient seemed to be more severely symptomatic than when having a common cold, but without fulfilling the criteria for pneumonia. Some GPs subdivided bronchitis into viral and bacterial. The decision between viral and bacterial depended, among other things, on the patient's comorbidities:

“So… most of the ones [cases of acute bronchitis] we see are viral…so I think I must differentiate […] there are typical risk factors […] you must think about that first. For me, there are viral and bacterial germs, and you must distinguish whether it is a COPD patient with a history of illness, or whether it is a young, fit patient who has bronchitis […] so I think you must subdivide again, but for me it [acute bronchitis] can be both.” (GP11).

The subdivision subsequently meant two different strands of action regarding therapy. The diagnosis “acute bronchitis” in contrast to other diagnoses in itself did not create a clear therapeutic decision. The term therefore did not serve as a key to prescribing an antibiotic or not. It seemed that, when being in the middle of estimated probabilities for viral or bacterial infection the decision crossed the tipping point toward antibiotic prescribing:

“Bronchitis…for me the probability that it is a bacterial infection rises to 50 per cent when I then think to myself: Ok, now I really do not know, is it already bacterial or not. So, when I write down this diagnosis, I give an antibiotic.” (GP1).

Other factors, for example the clinical context and comorbidities of the patients, came into play, guiding the decision:

“If I have a patient with high blood pressure or one who is cachectic, one who has diabetes and another disease history, who already had a little compliance before, then I would prescribe antibiotics to protect his body.” (GP4).

Other reasons named for antibiotic prescribing were increased inflammation markers like C-reactive protein (CRP), multimorbidity, doubts about good compliance or uncertainty in consultations on Friday evenings. Patients with comorbidities were suspected as having a higher risk for progression to pneumonia.

In mild cases, symptomatic therapies like mucolytics, painkillers or herbal remedies were preferred. The severity of the presented symptoms was seen as important, directly influencing the therapeutic decision:

“Mild bronchitis, without fever, […] can be treated quite well with expectorants, if you are not affected by general symptoms.” (GP12)

A lot of reasoning went into the details of symptoms. One characteristic that was used to distinguish between viral and bacterial infections was sputum color. The GPs connected a green or yellow color with bacterial infection, resulting in antibiotic prescribing:

“If there is [sputum] color, there is something bacterial in the background.” (GP5)

“And that was the most important question—he has a ‘greenish yellow’ sputum…there has been additionally a secondary infection on top, a second infection, which is bacterial.” (GP4).

Cough was described as “typical for acute bronchitis.” Productive cough was seen as a characteristic of acute bronchitis and seemed to be a presupposition for diagnosing it:

“Bronchitis is when there is a productive cough in the foreground. Often accompanied by headache, cold symptoms, but the cough is the leading symptom. With sputum most of the time, or more of an irritating cough at first becoming more productive as it progresses, so for me bronchitis is almost always productive.” (GP2).

Cough was also used to shape different stages of the process of the disease itself:

“If the symptoms and the coughing are paramount, then I would write down acute bronchitis […] Mild cough, is with little sputum, it is annoying but does not show any signs of general major illness, but the cough is simply there. The other stage is when we have sputum and hear a bronchial sound during auscultation, which is then stronger or clearer, moister, and coarse-bubbled. In the third stage, there is only a lack of vesicular breathing over the entire lung from top to bottom on both sides, there is simply bronchial breathing […].” (GP4).

Next to cough, symptoms of sinusitis, pharyngitis, common cold and otitis media were named, none of them being specific for acute bronchitis. Acute bronchitis was less perceived as a cognitively defined entity but as something that revealed itself in the overall clinical picture:

“Whereas it would really be my subjective impression, it's not what the patient says but how he appears to me […] if he simply appears to me to be sicker than a common cold—but I also don't hear anything above the lungs during auscultation, then I choose bronchitis…if I have the feeling that it could be something bacterial now, too. […] For me it is an embarrassing diagnosis, I have to say, so I take it when I cannot decide. Is it bacterial or not? That is exactly when I always take it.” (GP1).

#### The common cold and its differentiation from acute bronchitis

The common cold was conceptualized as a viral infection, where the patient usually is not very sick beyond annoying symptoms like a runny nose. In other words, the common cold is characterized by the absence of severe symptoms:

“The patient comes in and you can hardly tell that he is ill. They just say I am not feeling well but the patients look healthy, they have a good general condition—or a normal general condition and describe the symptoms to me. And you often do not find anything during examination but symptoms of a common cold, they say ‘my throat hurts,’ but you cannot really see exudates on the tonsils […] you don't really have any findings.” (GP1).

On the other hand, it was seen as a disease affecting the whole body. Cough was mentioned but played a minor role:

“Well, common cold for me is…more…the whole body, common cold is usually affecting the whole body, which means limb pain, headache and rhinitis, shivering, sour throat, and cough, but especially really affecting the whole body…where the whole body shows symptoms…and where cough plays a minor role.” (GP2).

Important was the low severity of the common cold. The common cold was much easier to diagnose for the GPs than acute bronchitis since its main symptoms are in the upper respiratory tract. Acute bronchitis was described as a "more" of a common cold and a consequence from it. With the common cold being conceptualized as of viral origin and rated overall as a mild disease, the decision against an antibiotic seemed to be clear and easy. Therapy for the common cold as for mild bronchitis was only symptomatic, using mucolytics, inhalation and resting.

#### Pneumonia and its differentiation from acute bronchitis

The definition of pneumonia seemed to be easier for the GPs. Again, the differentiation from the acute bronchitis was uncertain. Clinical symptoms with seemingly clear findings like tachypnea, dyspnea, fever, and a typical chest auscultation were listed:

“I have tachypnea, certain symptoms of dyspnea, a higher fever, a typical auscultation finding; I really have a worse general condition if I see a typical lobar pneumonia.” (GP11)

Fever was never mentioned as a single characteristic of pneumonia, but always in connection with other leading symptoms. According to the GPs, fever could also exist in patients with acute bronchitis. It was generally linked to a more aggressive infection, taking it as an alarm sign for severe disease and therefore lowering the threshold for antibiotic prescribing. As acute bronchitis was seen as an increase of the common cold, pneumonia was seen as an increase of acute bronchitis. It became clear that GPs compared again the severity and seriousness of the diseases:

“A classic pneumonia patient is in bad condition…Visually bad, heartrate is up, blood pressure is low, he is sweating, maybe a bit dizzy […] my feeling is that it is simply a level above bronchitis.” (GP2).

Overall, pneumonia was conceptualized as a severe and threatening disease.

The poor physical condition of patients with pneumonia imperatively led to prescribing an antibiotic to help with the patient's recovery. While the full recovery-time of acute bronchitis was estimated between one to three weeks, pneumonia was longer lasting and more dangerous for the patients, especially for older patients and for patients with other underlying conditions:

“It is the case that certain underlying conditions make the probability higher. This means that a patient who I know smokes has a higher probability of getting pneumonia, which is a very banal example, but it is relatively common. If I have someone who has asthma, they also know that they have a higher probability of getting pneumonia.” (GP3).

In vulnerable groups, the risk of a severe course was estimated to be significantly higher, increasing the willingness to prescribe antibiotics. Acute bronchitis and pneumonia seemed to exist in a direct interdependent relationship:

“If the patient has pneumonia, then he also has severe acute bronchitis.” (GP9)

Nevertheless, there still seemed to be a desire to differentiate the two diseases.

#### Role of technical diagnostics

One way to differentiate between acute respiratory tract infections, especially between pneumonia and acute bronchitis, were diagnostic tests like blood-tests and chest X-rays. The blood tests were used as a confirmation for the GPs’ working hypothesis, by differentiating between a viral and a bacterial infection and thereby reducing uncertainty. Even if often described as uneconomical, the desire for objective findings seemed to outweigh this negative aspect:

“You can take your stethoscope and auscultate. There are already indications of bronchitis itself…rales, although that can also be with pneumonia. (laughs) But with pneumonia, you already have a weakened breathing sound on one side, for example, and a high fever. But that can also be the case with bronchitis. Ultimately, only the blood tests and the X-ray make the clear differentiation.” (GP12).

The uncertainty of the distinction between acute bronchitis and pneumonia leads to a lack of confidence in one's clinical examination. Therefore, the attempt was made to compensate for this uncertainty by using technical and supposedly objective diagnostics. Chest X-Ray was described to only be used in patients with the suspected diagnosis of pneumonia. It was seen as a good but rarely as an accessible diagnostic tool:

“It would play a bigger role if we would get appointments. The last patient with pneumonia I had a few days ago…did get an appointment after four weeks. Therefore, we are not able to do chest X-ray according to the guidelines and that is a real detriment situation for us.” (GP3).

#### Diagnostic uncertainty and gut feelings

Diagnostic uncertainty, gut feelings, and recognition of an overall clinical picture (gestalt) were prominent in every interview. GPs had difficulties to describe how uncertainty or gut feelings influence their decisions. The pressure of having to end up with the decision of prescribing an antibiotic or not, forced the GPs to frequently decide under conditions of uncertainty:

“You must treat 100 percent of the patients even though you only know the right diagnosis of a small part of them in the beginning.” (GP2).

In this process gut feeling came into play by stating things like *“simply knowing the story of the patient”* and a *“certain feeling.”* If that feeling was not good, there was a tendency to prescribe antibiotics:

“It’s the first impression, when the patient enters the room […] If I don’t have the right feeling, then something is wrong.” (GP10)

2. ***Patients’ expectations concerning therapy***

GPs reported that patients’ expectations put pressure on them during the consultation. The pressure was perceived especially regarding antibiotic prescribing. GPs usually met the patient’s demands with conversation and educational efforts about mechanisms of action. Consensus was usually reached through shared-decision-making or delayed prescribing of an antibiotic. In some cases, however, it also became clear how the GPs gave in to the patient’s wishes to protect the doctor-patient-relationship, due to lack of time or because of a lack of personal resilience:

“Because at some point he says, I have had enough, I have been coughing for three weeks now, I want to get well again. What option do I have? So, if I have already given him cough syrup for two to three weeks, he will not accept the next cough syrup amicably.” (GP6).

It repeatedly seemed that curing was placed above caring, and a fast recovery of the patient was stated as the highest goal. In respiratory tract infections, this again led to the prescribing of antibiotics.

3. ***Antibiotic prescribing in respiratory tract infections and the concept of bacterial secondary infection***

Antibiotic prescribing or non-prescribing was seen as the central question in treating respiratory tract infections. The antibiotic was mentioned when both doctor and patient felt they were at a point where now something effective must be done. It seemed as if the antibiotic is seen by patients and GPs alike as an effective therapy, not considering its individual indication.

Particularly in patients with comorbidity, the GPs were warier of the patient getting a secondary bacterial infection. Here, the antibiotic was even considered to have greater effects.

The secondary bacterial infection coming on top of an initially viral disease was an important part of the concept of acute bronchitis, serving as an explanation and justification for prescribing antibiotics in the reasoning process. There was even seen as a preemptive indication for prescribing antibiotics, based on pathophysiologic reasoning:

“So, my concept [of the secondary bacterial infection] is that the patient is weakened. The mucous membranes are attacked, and this simply makes it easier for bacteria to colonize and multiply and make the whole thing worse. Yes, exactly, and with the antibiotic I can possibly avoid an additional bacterial infection if the patient’s general condition is already precarious.” (GP12).

In addition to that, the harm of prescribing antibiotics, even if judged as unnecessary was rated low.

“[…] then you also think for yourself, is it now bad if I give it [the antibiotic] to him although I do not think that he needs it? And sometimes I then think to myself you just give an Amoxicillin, and it will not really harm him. Not the state of the art but you can give it a try.” (GP2).

## Discussion

### Main findings

GPs conceptualize acute bronchitis with a variety of uncertain symptoms with productive cough being in the center of it. It is categorized between common cold and pneumonia with undetermined borders and labels and is diagnosed when neither of the two seems to fit well as the correct diagnosis. The fuzziness of the concept “acute bronchitis” seems to correspond well to the diagnostic crossroads and the uncertainty GPs frequently are in. While from most diagnoses a definite treatment follows, this does not seem to be the case with acute bronchitis. The diagnosis leaves the GP in uncertainty of whether to prescribe an antibiotic or not. Other factors then seem to tip the balance in the direction of antibiotics. Antibiotics are used to treat or to protect patients from severe complications. Prescribing is based on a mixture of comorbidities, gut feelings and pathophysiologic reasoning often recognized by certain clinical signs like colored sputum. The expectation of a faster recovery time, the concept of avoiding a secondary bacterial infection, and patient expectations play important roles for antibiotic prescribing.

### Comparison with existing literature

Concepts are built upon personal experiences, tacit or explicit knowledge and shared assumptions and conventions. Not only for acute bronchitis, they mostly seem to be non-explicit, fuzzy, and variable between individuals [27]. Terms, concepts and classifications behind them, are applied to describe reality and to order human interaction [28]. The mutual exclusiveness of categories created by definitions of disease concepts in classifications is hardly matching to clinical reasoning and decision making. Guidelines are meant to support and guide clinical decisions, competing with implicit “mindlines” [29]. Gabbay et al. conducted a qualitative study with primary care clinicians in England and described similar findings to ours, where decision-making could not be explained properly, but seemed to be more a multifactorial construct consisting of expert knowledge and gut feeling than on literature, guidelines, and explicit concepts [29]. In 2000, Hueston asked whether the diagnosis of acute bronchitis, due to its only marginal differences to the common cold, should exist any longer as it frequently leads to antibiotic overprescribing [30]. Nevertheless, patients are still labelled as having acute bronchitis. It seems reasonable to apply a fuzzy concept in a situation of high uncertainty. Corresponding well to the literature, productive cough was identified as one of the most important reasons for applying the diagnosis of acute bronchitis in our study and affecting the prescribing of antibiotics [30, 31]. The most important clinical sign used for discriminating between a viral or a bacterial origin of the infection that could be identified in our study was sputum color. Although studies have shown that sputum color cannot define whether an infection is bacterial or viral (in non-COPD patients), the majority of our interviewed GPs associated sputum color with a secondary bacterial infection [32, 33].

Secondary bacterial infection was a major element in the mostly pathophysiological clinical reasoning of our participants, which was also seen in other studies [34]. The theory behind this is not supported by clinical evidence, at least not as a justification for antibiotic prescribing. Mechanisms proposed are a virus-caused low immunologic defense that allows bacteria to colonize [35]. In fact, it was shown for influenza that patients have reduced mucociliar clearance which enables pneumococci to attach better [36, 37]. The knowledge of these mechanisms and facts, even if being improbable in most cases, might explain the GP’s fear of missing a serious diagnosis or a complication. This fear may lead to accepting a possibly unnecessary therapy [36].

Corresponding to our results, Dempsey et al. reported patients’ expectations being a major reason for antibiotic prescribing [38]. Even if GPs’ perceptions of their patient’s expectations do not necessarily correspond to what patients really expect, this perception influenced the resulting therapy [10, 11]. Colliers et al. described this divergence with the fact that expectations in doctor-patient-relationships are often not verbalized [9]. Therefore, the importance of assumptions about the expectation of the other increases strongly and the communication becomes an essential tool for dealing with unnecessary antibiotic prescriptions [9]. A study in Bavaria reported that GPs trusted their decisions on antibiotics, underestimated the influence of patient expectations and at the same time wanted more medical training [39]. It might be possible that these results reflect the uncertainty of the GPs, of which they are sometimes not aware themselves [39]. A study from Netherlands identified point of care testing and medical training with GPs with lower antibiotic prescription rate in respiratory tract infections [40]. Here, also one reason for this finding might be the reduction of the GPs uncertainty.

### Implications for practice and research

In theory, diagnostic categories can be defined and clearly distinguished. In practice this is often not the case. We found acute bronchitis being diagnosed when there is an uncertain diagnose between the common cold where antibiotics are clearly not indicated and pneumonia where they are. In this space, theory seems to betray the practitioners. A famous old saying goes that “In theory, theory and practice are the same. In practice, they are not.” Theory hardly leaves room for uncertainty. We as researchers tend to assume that theory is right, and practice is not. Theory mostly falls short in the complexity, variability, and the resulting uncertainty of clinical situations in the *“swampy lowlands”* [41] of the practice of medicine.

In practice we must decide under conditions of uncertainty. Ludmerer once claimed: *“…the failure to educate physicians about uncertainty was ‘the greatest deficiency of medical education throughout the twentieth century’*.” [42] Correcting this deficiency might help GPs deciding against antibiotic prescribing with more confidence. In his article “The ethics of uncertainty” Djulbegovic claims that it was Evidence-based Medicine that brought the notion of uncertainty into medical thinking [43]. Focusing medical training, vocational training and continuous medical education more on probabilistic reasoning and the principles of Evidence-based Medicine than on pathophysiologic reasoning might be helpful [44, 45].

### Limitations and strengths

We recruited only a small number of GPs in the surroundings of Erlangen, Bavaria. Patient care and GP’s ideas might be different in bigger cities or rural areas of Germany, and more in other countries. For our qualitative study, there was no need for representativity at this point. As saturation of information was reached, the number of the interviews remained small. This made it possible to stay focused, extracting the most important points of the interviews and building a base for further research. We see a strength of our study in that we used the documentary method for analysis. It is a method of reconstructive social research, which is rather unusual for general practice but enabled us to approach the research topic in a new and unconventional way. Partially, during the interviews, it could be seen that the GPs seemed to be under pressure to give the “right” answers. Although they were informed before, that there is no judgement of their statements, the presence of an interviewer, being a student from the university, might have influenced their statements during the interviews. The distortion of answers might be bidirectional. On one hand being confronted with someone from the university, the presumed stronghold of theory, might have intimidated the practitioners. On the other hand, the fact that they had a student in front of them might have contributed to openness that allowed talking about issues like diagnostic uncertainty in the first place.

## Conclusion

GPs apply the term acute bronchitis when they are highly uncertain of whether antibiotic prescribing is indicated for a respiratory infection or not. The fuzzy concept of the acute bronchitis seems to match the uncertainty of the clinical situation. Moreover, in contrast to most other diagnoses, no clear treatment pathway emerges from the concept. In this uncertain situation, many other factors such as the fear of secondary bacterial infection come into play, tipping the balance towards prescribing antibiotics. Teaching students, physicians in vocational training and continuous medical education to better deal with uncertainty might help reducing antibiotic overprescribing in the future.

## Supplementary Information

**Additional file 1.**

**Additional file 2.**

## Data Availability

The datasets (anonymized interview transcripts) used and analysed during the current study are available from the corresponding author on reasonable request.

## Abbreviations

GP: General practitioner
