# Supplementary material for: The conceptualization of acute bronchitis in general practice – a fuzzy problem with consequences? A qualitative study in primary care
Source: BMC Prim Care. 2023 Apr 6;24:92. doi: 10.1186/s12875-023-02039-z (PMC10080804; doi:10.1186/s12875-023-02039-z)
Supplement: Supplementary file 1 — Additional file 1. [file 12875_2023_2039_MOESM1_ESM.pdf]

Interview guide: Conceptualization of acute bronchitis

Opening question:
" Thinking about your last patient with bronchitis, please tell me the course of the consultation?"

- How did the consultation go?
- How did you feel about it?
- Difficulties/uncertainties in giving the diagnosis?
- How often do you see patients with acute bronchitis?

How do you work with the three diagnoses: Common cold, acute bronchitis and pneumonia?

- Personal definition of common cold/ acute bronchitis/ pneumonia?
- Differentiation of the three diagnoses from each other?
- Clinic/pathogen/medication/diagnosis (sputum color, fever, chest X-rays)

  ➔ Goal: Clarification of the differentiation of the diagnoses: Acute bronchitis, common cold and pneumonia.

- What do you normally prescribe for the diagnoses mentioned above?
- How often do you see patients diagnosed with acute bronchitis?
- How threatening do you consider the course of the diseases to be with/without therapy/cure?

What role do patient expectations play?

- What do patients expect from you?
- …regarding antibiotics?
- What kind of disease concept do they have in mind?

Side questions:

- There are data that say that antibiotics are prescribed even though the patient has a common cold (although no benefit is proven); How would you explain this? How is the fear of not finding pneumonia affecting the treatment of acute bronchitis?
- What role does diagnostic uncertainty play?
- The guideline "Cough" of the German Society for General and Family Medicine (DEGAM) advises against prescribing antibiotics for the diagnosis of "acute bronchitis" because at least 80% of cases are viral infections. At the same time, it nevertheless recommends the use of antibiotics in patients with chronic concomitant diseases or immunosuppression. Viruses do not react to antibiotics even in chronically ill patients. How do you explain this recommendation of the DEGAM[1]?
- Let's assume it is infection time, your waiting room is full of patients, and you have the symptoms described at the beginning yourself. Which therapy would you choose for yourself?

Thank you very much for the interview.

[1] Note: Status of the guideline at the time of the interviews. The cited guideline has been revised 2021; in the current version, antibiotics are generally no longer recommended for acute bronchitis, regardless of the viral or bacterial genesis of the disease. Antibiotic therapy is reserved in acute bronchitis for those patients who cannot be differentiated from pneumonia due to severe pre-existing conditions.
