# Supplementary material for: The conceptualization of acute bronchitis in general practice – a fuzzy problem with consequences? A qualitative study in primary care
Source: BMC Prim Care. 2023 Apr 6;24:92. doi: 10.1186/s12875-023-02039-z (PMC10080804; doi:10.1186/s12875-023-02039-z)
Supplement: Supplementary file 2 — Additional file 2. [file 12875_2023_2039_MOESM2_ESM.pdf]

Uniklinikum Erlangen

Allgemeinmedizinisches Institut
Universitätsstr. 29
91054 Erlangen

Questionnaire for participating GP practices

Dear Colleagues,

thank you for participating in a project of the Department of General Medicine at the University Hospital Erlangen regarding the question "Conceptualization of acute bronchitis". The basic information obtained throughout the questionnaire allows us to describe the surveyed sample in outline. The questionnaire is filled out anonymously and it is therefore not necessary to provide personal data.
Of course, the information provided will be treated strictly confidential.
For the sake of better readability, no distinction has been made between male and female spelling. The respective designation always refers to all genders.

Please note the following when completing the form:

- Please do not enter any personal data (name/date of birth or similar)
- Print in the free text areas
- Tick the boxes clearly: X
  In case of accidental wrong selection, please fill in the corresponding box completely and reenter your correct selection next to the box

Please return the completed questionnaire to the interviewer only once the interview is completed.

With many thanks for your participation,

Nadine Schubert und Prof. Dr. Thomas Kühlein

Friedrich-Alexander-Universität Erlangen
Allgemeinmedizinisches Institut
Universitätsstraße 29
91054 Erlangen
Germany

Questionnaire for participating GP practices

Where is your practice located?

☐ City radius 20 km

☐ Rural area

In which form of practice do you work?

☐ Single Office ☐ Joint Practice

☐ Group Practice ☐ Medical Care Center

What is the average number of patients in your practice?

☐ < 500 patients ☐ 500-1000 patients

☐ 1001-1500 patients ☐ > 1500 patients

How many colleagues work in the practice besides you?

☐ Single Office Number of Colleagues: ___

Your Birthyear **19** ___ (Please fill in the year)

What is your sex? ☐ female ☐ male

What year did you establish your practice? ___

(Please fill in the year)

What is your specialty?

☐ General practitioner

☐ GP Internist ☐ Others: ___
